# Nanoscale-Resistive Switching in Forming-Free Zinc Oxide Memristive Structures

**DOI:** 10.3390/nano12030455

**Published:** 2022-01-28

**Authors:** Roman V. Tominov, Zakhar E. Vakulov, Nikita V. Polupanov, Aleksandr V. Saenko, Vadim I. Avilov, Oleg A. Ageev, Vladimir A. Smirnov

**Affiliations:** 1 Department of Radioelectronics and Nanoelectronics, Institute of Nanotechnologies, Electronics and Electronic Equipment Engineering, Southern Federal University, 347922 Taganrog, Russia; tominov@sfedu.ru (R.V.T.); avsaenko@sfedu.ru (A.V.S.); 2 Federal Research Centre the Southern Scientific Centre of the Russian Academy of Sciences, 344006 Rostov-on-Don, Russia; vakulov@ssc-ras.ru; 3 Laboratory of Functional Nanomaterials Technology, Southern Federal University, 347922 Taganrog, Russia; npolupanov@sfedu.ru; 4 Department of Micro- and Nanoelectronics, Institute of Nanotechnologies, Electronics and Electronic Equipment Engineering, Southern Federal University, 347922 Taganrog, Russia; avilovvi@sfedu.ru (V.I.A.); ageev@sfedu.ru (O.A.A.)

**Keywords:** neuromorphic systems, memristor, ReRAM, resistive switching, forming-free nanocrystalline ZnO, pulsed laser deposition

## Abstract

This article presents the results of experimental studies of the impact of electrode material and the effect of nanoscale film thickness on the resistive switching in forming-free nanocrystalline ZnO films grown by pulsed laser deposition. It was demonstrated that the nanocrystalline ZnO film with TiN, Pt, ZnO:In, and ZnO:Pd bottom electrodes exhibits a nonlinear bipolar effect of forming-free resistive switching. The sample with Pt showed the highest resistance values *R_HRS_* and *R_LRS_* and the highest value of *U_set_* = 2.7 ± 0.4 V. The samples with the ZnO:In and ZnO:Pd bottom electrode showed the lowest *U_set_* and *U_res_* values. An increase in the number of laser pulses from 1000 to 5000 was shown to lead to an increase in the thickness of the nanocrystalline ZnO film from 7.2 ± 2.5 nm to 53.6 ± 18.3 nm. The dependence of electrophysical parameters (electron concentration, electron mobility, and resistivity) on the thickness of the forming-free nanocrystalline ZnO film for the TiN/ZnO/W structure was investigated. The endurance test and homogeneity test for TiN/ZnO/W structures were performed. The structure Al_2_O_3_/TiN/ZnO/W with a nanocrystalline ZnO thickness 41.2 ± 9.7 nm was shown to be preferable for the manufacture of ReRAM and memristive neuromorphic systems due to the highest value of *R_HRS_*/*R_LRS_* = 2307.8 ± 166.4 and low values of *U_set_* = 1.9 ± 0.2 V and *U_res_* = −1.3 ± 0.5 V. It was demonstrated that the use of the TiN top electrode in the Al_2_O_3_/TiN/ZnO memristor structure allowed for the reduction in *U_set_* and *Ures* and the increase in the *R_HRS_*/*R_LRS_* ratio. The results obtained can be used in the manufacturing of resistive-switching nanoscale devices for neuromorphic computing based on the forming-free nanocrystalline ZnO oxide films.

## 1. Introduction

Over the past few decades, the information-technology market has grown rapidly, largely because the performance of computing systems has improved significantly over time due to Moore’s law [1,2,3,4]. Big-data processing is considered a new benchmark in addition to performance, which is of great importance in applications such as the Internet of Things (IoT), autonomous vehicles, and adaptive control and management systems [5,6]. Currently, further increases in the efficiency of big-data processing are limited by the resolution and the cost of manufacturing complementary metal oxide semiconductor (CMOS) transistors less than 10 nanometers in size, creating the difficulty of sustainable and cost-effective scaling [7,8]. Therefore, there is a need to develop and research a new base of non-volatile elements of electronics with increased computational efficiency, which would meet the requirements of the information-technology market [9,10,11,12,13]. 

Memristor (ReRAM) is a memory and computing device that has appeared in the last few years and has shown great promise as a new-generation universal memory capable of operating in both digital and analog modes [14,15]. The advantages of ReRAM include non-volatility, low power consumption, high speed, small dimensions (~10 nm), as well as low manufacturing costs, and the ability to integrate with CMOS technology [16,17]. It should be noted that there is a theoretical possibility of reducing the ReRAM memory element to the atomic level, which will further improve the characteristics of the resistive switching effect by improving the uniformity of the nanoscale conduction channel, as well as reducing the current flowing through the ReRAM memory element, which will lead to a further decrease in power consumption [18,19,20,21,22,23,24,25,26,27,28,29].

The resistive switching effect is exhibited in many classes of materials, such as chalcogenides, perovskites, organic compounds, solid state electrolytes, graphene, and metal oxides [30,31,32,33,34,35,36,37,38,39,40]. Among the latter are binary oxides such as ZnO, HfO_2_, ZrO_2_, NiO, TiO_2_, WO_3_, TaO_x_, and Gd_2_O_3_, which is of particular interest due to its compatibility with CMOS technology, multi-bit switching, and simple chemistry [41,42,43,44,45,46,47,48]. ZnO-based devices are used to simulate biological neurons and synaptic functions [49,50]. One of the main problems in the manufacture of ReRAM is obtaining controlled characteristics of switching behavior and electrical properties. From a semiconductor physics point of view, the metal/oxide interface and oxide thickness are key factors in determining the formation of oxygen vacancies and charge transport. Therefore, it is important to study the influence of geometric and electrophysical parameters, as well as the influence of the electrode material and their doping on the values and range of resistances of the high resistance state (*R_HRS_*) and the low resistance state (*R_LRS_*) for the manufacture of ReRAM elements of energy-efficient neuromorphic systems operating at low switching voltages (*U_set_* and *U_res_*) [51,52,53,54,55,56,57]. Despite several works reporting the observation of resistive switching in ZnO, there is still a lack of knowledge about various aspects for the manufacture of elements of memristive neuromorphic systems based on this material.

There are many metal-oxide film-fabrication methods: atomic layer deposition (ALD) [58,59], the sol-gel method [60], magnetron sputtering [61,62], and pulsed laser deposition (PLD) [63]. Forming-free nanocrystalline ZnO films grown using PLD have been shown to exhibit improved resistive switching characteristics, with higher *R_HRS_*/*R_LRS_* ratios and lower operating voltages [64,65,66,67]. PLD is a relatively inexpensive technology for growing thin films that meets the requirements for fabricating memristive devices, primarily because of the possibility of precise adjustment of the electrophysical parameters of the oxide by varying the growth parameters.

In this work, we experimentally investigated the influence of the bottom electrode (BE) material and the thickness of the forming-free nanocrystalline ZnO film on the resistances of the *R_HRS_*, *R_LRS_,* and *R_HRS_*/*R_LRS_*, as well as on the switching voltages *U_set_* and *U_res_*. Then, nanoscale resistive switching study of forming-free nanocrystalline ZnO films was represented using conductive atomic force microscopy (CAFM). The influence of PLD growth conditions on the electrophysical and morphological parameters of the obtained films was investigated. In the end, we fabricated and investigated forming-free resistive switching in Al_2_O_3_/TiN/ZnO/TiN memristive structures. We considered that the new results obtained in the article will be useful to readers and can be used in the development of neuromorphic systems based on memristor structures and zinc oxide.

## 2. Materials and Methods

To investigate the effect of the bottom-electrode material on resistive switching in the nanocrystalline ZnO film, samples with different bottom-electrode materials (TiN, Pt, ZnO:In, ZnO:Pd) were prepared using a Pioneer 180 PLD module (Neocera LCC, Beltsville, MD, USA) with a KrF excimer laser (*λ* = 248 nm). Sapphire wafer (Al_2_O_3_) was used as a substrate. An high-energy electron-diffraction system (k-Space Associates Inc., Dexter, MI, USA) entering the composition of the PLD module was used for RHEED study. The lattice constant was determined using the kSA-400 (Version 1.0) software. The bottom electrodes were grown under the following conditions: substrate temperature: 500 °C; target–wafer distance: 50 mm; background pressure (vacuum): 10^−5^ Torr; pulse energy: 400 mJ; laser-pulse repetition rate: 10 Hz; and number of laser pulses: 10,000. Then, on the bottom electrodes of all samples in a single vacuum cycle, a nanocrystalline ZnO film was deposited on the PLD under the following conditions: substrate temperature: 450 °C; target–substrate distance: 60 mm; oxygen pressure: 0.5 mTorr; pulse energy: 400 mJ; laser-pulse repetition rate: 10 Hz; number of laser pulses: 3000. All nanocrystalline ZnO films were annealed in a nitrogen atmosphere with a pressure of 10^−3^ Torr at a temperature of 1200 °C for 10 h for forming free resistive switching [64].

For electrical measurements, the nanocrystalline ZnO films were deposited through a special mask pattern to provide access to the bottom electrode. Thus, the film was not deposited around the bottom electrode protected by the mask.

The structure of ZnO films was studied by X-ray diffractometry (XRD) using a Rigaku Miniflex 600 diffractometer (Rigaku Corporation, Tokyo, Japan).

The temperature dependences of the change in Gibbs free energy (Ellingham diagram) were calculated using the FactSage 6.2 software package for chemical-reaction analysis (GTT-Technologies, Herzogenrath, Germany) with an updated electronic database of the temperature dependence of thermophysical parameters.

To study the nanoscale-resistive switching in forming-free ZnO films, we used a conductive atomic force microscopy (CAFM) mode on the Ntegra Probe Nanolaboratory (NT-MDT, Zelenograd, Russia). For this aim, the method described in [64,65] was used. As a result, CAFM images of the zinc oxide resistive switching were obtained under the following regimes: HRS spot at +5 V, LRS spot at −5 V, and CAFM reading voltage +1 V. Spots were investigated during 10^4^ s. Based on the results obtained, the retention test was plotted.

Electric measurements were carried out using a semiconductor characterization system Keithley 4200-SCS (Keithley Instruments, Solon, OH, USA) and an EM-6070A submicrometer probe system (Planar, Minsk, Belarus) with 100 nm tip diameter tungsten (W) probes. The bottom electrode was grounded for all samples. To prevent thermal breakdown of the nanocrystalline ZnO film, a compliance current of 10^−4^ A was set during the electric measurements. As a result, the current-voltage curves (CVC) were obtained at a voltage sweep of −3 to +3 for 10 cycles for each sample at the same point on the ZnO surface. The read voltage was 0.5 V. Curve analysis was implemented using Origin 8.1 software (OriginLab Corporation, Northampton, MA, USA). Based on the results obtained, the resistances *R_HRS_*, *R_LRS_*, *R_HRS_/R_LRS_*, *U_set_,* and *U_res_* were evaluated.

The thickness of the nanocrystalline ZnO film was investigated by measuring the height of the TiN/ZnO “step” made by photolithography. For this, Al_2_O_3_/TiN/ZnO samples were prepared with different numbers of laser pulses for ZnO from 1000 to 5000. Then, the FP-383 photoresist was applied to the ZnO film by the centrifugation method at a rotation speed of 4000 rpm. The photoresist film was exposed through a photomask to UV radiation for 2 min, developed in a 5% aqueous solution of KOH, and tanned at 110 °C for 20 min. Areas of the ZnO film that were not protected by photoresist were etched in an ammonium hydroxide/water solution (1:2) for 30 s. Photoresist residues were removed with dimethylformamide. The step height (Figure S1) and the surface roughness were investigated using the Ntegra Probe Nanolaboratory in semi-contact AFM mode using NSG11 cantilevers. AFM scanning was carried out with the following parameters: velocity: 80 μm/s; scan area: 1 × 1 μm^2^; and SetPoint: 7 nA. AFM image processing was performed using Image Analysis software. To confirm the AFM results, we also investigated the nanocrystalline ZnO film surface using a Nova NanoLab 600 scanning electron microscope (FEI Company, Hillsboro, OR, USA). SEM images were obtained with the following parameters: acceleration voltage: 10 kV and magnification: 300,000×. Based on the experimental results obtained, the dependence of the nanocrystalline ZnO film thickness and surface roughness on the number of laser pulses were tabled.

The electrophysical parameters of the nanocrystalline ZnO films were studied using an Ecopia HMS-3000 Hall effect system (Ecopia Co., Anyang, Korea). The dependences of the electron concentration, electron mobility, and resistivity of the nanocrystalline ZnO films on their thicknesses were plotted. The study of the influence of the thickness of the nanocrystalline ZnO film on resistive switching in the Al_2_O_3_/TiN/ZnO structure was also carried out with the Keithley 4200-SCS semiconductor characterization system. For this purpose, the CVCs were measured at the same point on the ZnO surface at voltage sweeps of −3 to +3 for each sample. The read voltage was 0.5 V. Based on the obtained results, the *R_HRS_*, *R_LRS_,* and *R_HRS_/R_LRS_* dependences of the Al_2_O_3_/TiN/ZnO structures on the thickness of the forming-free nanocrystalline ZnO film were plotted. Furthermore, the dependence of *R_HRSp_*, *R_LRSp_,* and *R_HRSp_*/*R_LRSp_* on the switching cycle number (endurance test) was plotted. To study the effect of resistive switching at different points on the ZnO surface (homogeneity test), CVCs were obtained at different points on the surface of the forming-free nanocrystalline ZnO film on each sample. Based on the results obtained, *R_HRSs_*, *R_LRSs_,* and *R_HRSs_*/*R_LRSs_* at different points on the surface of the ZnO film of each sample were determined and tabled.

To study the influence of the top electrode on the effect of resistive switching, Al_2_O_3_/TiN/ZnO/TiN memristive structures (16 samples) were fabricated. The film of the top electrode was grown by PLD in regimes identical to those of the bottom electrode. The CVCs were also measured at voltage sweeps of −4 to +4 V for each sample. The read voltage was 0.5 V. Based on the results obtained, the endurance test and device-to-device reproducibility were plotted.

To study the influence of the amplitude pulse voltage (*U_A_*) and the pulse time duration (*t_A_*) on the *R_HRS_/R_LRS_* ratio, an endurance test was carried out in the range of 3 to 7 V and 20 to 60 ms for 1000 cycles for each of the *U_A_* and *t_A_* values, respectively. Based on the experimental results obtained, the dependences of the *R_HRS_/R_LRS_* ratio on *U_A_* and *t_A_* were plotted.

## 3. Results

**Figure 1:**
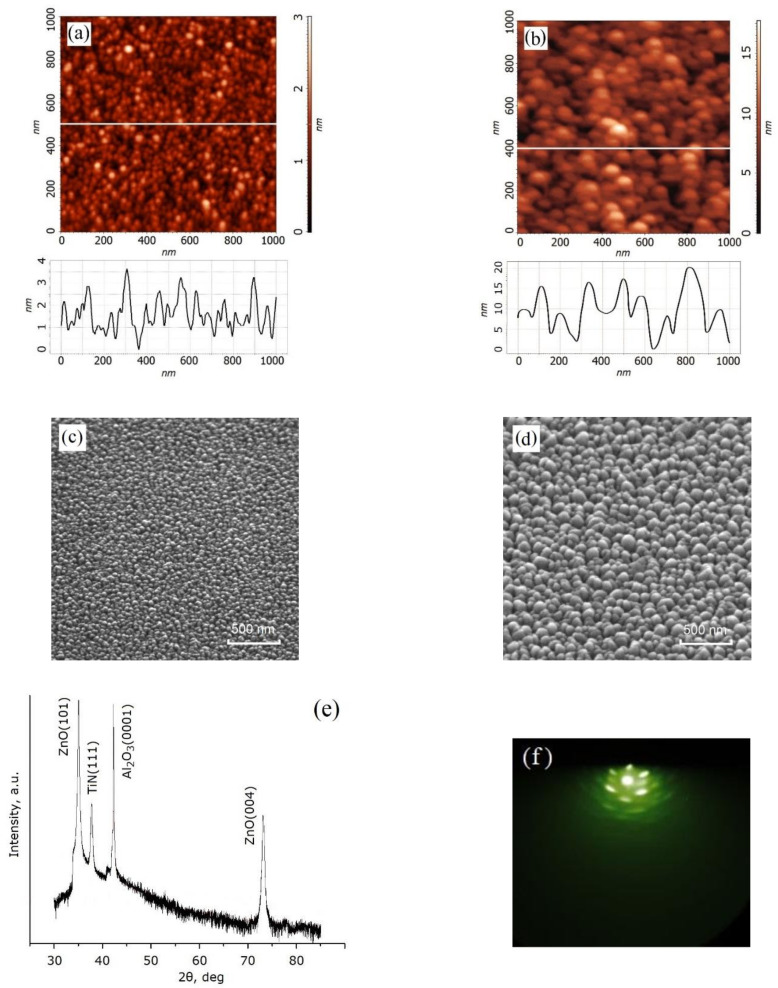
Nanocrystalline ZnO films surface at 1000 (**a**,**c**,**e**) and 5000 (**b**,**d**,**f**) number of laser pulses: (**a**,**b**)—AFM-image and AFM cross-section along the white line; (**c**,**d**)—SEM images; (**e**)—XRD spectrum of the Al_2_O_3_/TiN/ZnO structure; (**f**)—RHEED pattern.

Figure 1a–d shows the morphology of the zinc-oxide film. An increase in the number of laser pulses from 1000 to 5000 was shown to lead to an increase in surface roughness from 3.1 ± 0.7 nm to 19.8 ± 3.5 nm. The XRD spectrum of Al_2_O_3_/TiN/ZnO was characterized by a pronounced peak of ZnO (101) at 34° and ZnO (004) at 74° (Figure 1e). The lattice constant was established to be equal to 0.31 nm (Figure 1f). Thus, it was shown that the zinc-oxide film studied had a nanocrystalline structure.

**Figure 2:**
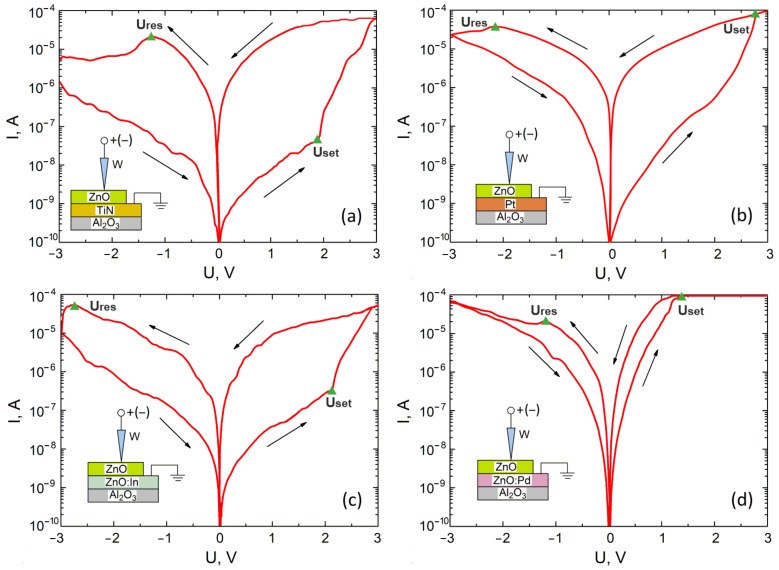
Current–voltage characteristics of the forming-free nanocrystalline ZnO films with various bottom-electrode materials: (**a**)—TiN; (**b**)—Pt; (**c**)—ZnO:In; (**d**)—ZnO:Pd.

An analysis of the results showed that a nanocrystalline ZnO film with a bottom electrode of TiN, Pt, and ZnO:In and ZnO:Pd exhibits non-linear bipolar forming-free resistive switching, according to which the electric field gradient and the kinetic energy of the electrons prevail over the concentration gradient and the temperature gradient [16] (Figure 2).

The values obtained for the *R_HRS_* and *R_LRS_* resistances, the *R_HRS_*/*R_LRS_* ratio, and the switching voltages *U_set_* and *U_res_* are shown in Table 1. It was shown that the highest ratio *R_HRS_/R_LRS_* = 2307.8 ± 166.4 was possessed by a sample with a TiN bottom electrode. The sample with Pt shows high resistance, *R_HRS_* and *R_LRS_* values, and the highest value of *U_set_* = 2.7 ± 0.4 V. The samples with bottom electrodes made of ZnO:In and ZnO:Pd showed lower values of the *R_HRS_* and *RLRS* and the *R_HRS_/R_LRS_* ratio, compared to those of TiN and Pt, but were more stable than *U_set_* and *U_res_*. The sample with the ZnO:Pd bottom electrode, in this case, showed the lowest *U_set_* = 1.2 ± 0.1 V.

**Figure 3:**
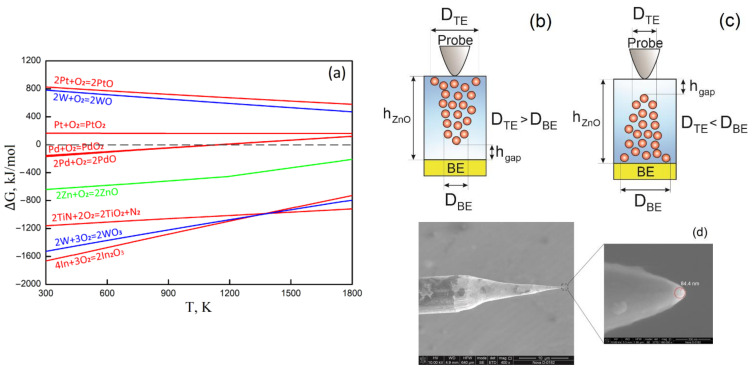
Electrode/oxide boundary influence on the resistive switching in the nanocrystalline ZnO film: (**a**)—Ellingham diagram; (**b**,**c**)—BE/ZnO/W structure scheme of resistive switching in the vicinity of the top electrode (**b**) and bottom electrode (**c**); (**d**)—SEM image of the top W electrode.

To explain the results obtained, an Ellingham diagram (Figure 3a) was built for the metal oxides formed due to the reduction reaction at the interface between the electrode and the forming-free nanocrystalline ZnO film (Equations (S1)–(S9)).

The interface between a metal electrode and an oxide is known to play a key role in resistive switching, especially for bipolar switching. The oxygen vacancies resulting from the redox reaction determine the potential barrier at the metal/oxide interface [61,62]. Bipolar switching is common for asymmetric structures. Different materials of the top and bottom electrodes lead to the fact that the interface with many oxygen vacancies serves as a reservoir (the base of the nanoscale conduction channel); switching occurs at the opposite interface with a smaller number of oxygen vacancies (the apex of the nanoscale conduction channel). A rectifying contact is observed at interfaces with a small number of oxygen vacancies; an increase in the number of oxygen vacancies leads to ohmic conductivity. In turn, the number of oxygen vacancies is determined by the Gibbs free energy change (∆*G*), an increase that leads to an increase in the number of oxygen vacancies, and a decrease that leads to a decrease in the number of oxygen vacancies.

Analysis of the Ellingham diagram showed that the ∆*G* curve for ZnO + O_2_ is higher than the ∆*G* curve for TiN + O_2_ and W + O_2_ (Figure 3a). This means that both the TiN/ZnO and ZnO/W interfaces are sources of oxygen vacancies. At the same time, because the ∆*G* curve for TiN + O_2_ is higher than W + O_2_ (Equation (S7)), it can be assumed that more oxygen vacancies are concentrated at the ZnO/W interface than at TiN/ZnO, that is, the base diameter is greater than the diameter of the apex of the nanoscale conduction channel (*D_TE_* > *D_BE_*) (Figure 3b). The reaction that results in the formation of oxide WO_3_ (Equation (S8)) does not proceed, since its curve ∆*G* > 0 kJ/mol. Therefore, it can be assumed that, with high probability, the generation and recombination of oxygen vacancies in the TiN/ZnO/W structure, i.e., resistive switching, occurs at the TiN/ZnO interface (Figure 3b). Both ∆*G* curves for Pt + O_2_ (Equations (S2) and (S3)) are located above 0 kJ/mol, which in theory implies the absence of a reduction reaction with the formation of oxygen vacancies at the Pt/ZnO interface. Therefore, with a high probability in the Pt/ZnO/W structure, *D_TE_* > *D_BE_* and resistive switching occurs at the Pt/ZnO interface (Figure 3b).

The ∆*G* curve for In + O_2_ (Equation (S4)) is located lower than for W + O_2_ and Zn + O_2_; however, taking into account the fact that it is a doping component and that the number of its atoms is clearly less than the number of W atoms, as well as the fact that ∆*G* for In + O_2_ and W + O_2_ (Equation (S7)) are approximately the same, then resistive switching in the ZnO:In/ZnO/W structure can occur both at the ZnO:In/ZnO interface (Figure 3b) and at the ZnO/W interface (Figure 3c). To minimize the probability of the generation of two or more nanoscale conduction channels, we used a tungsten probe with a tip radius of 84.4 nm (Figure 3d).

The ∆*G* curves for Pd + O_2_ (Equations (S5) and (S6)) are located near 0 kJ/mol, significantly higher than the ∆*G* curve for W + O_2_ (Equation (S7)). Taking into account the fact that Pd is a dopant and the number of Pd atoms is clearly less than the number of W atoms, as well as the fact that the ∆*G* values for W + O_2_ are positive at temperatures above 1138 K (Figure 3a), then, with high probability, we can assume that resistive switching occurs at the ZnO:Pd/ZnO/W structure at the ZnO:Pd/ZnO interface (Figure 3c).

Analysis of the Ellingham diagram showed that the ∆*G* curve for TiN + O_2_ is lower than for Pt + O_2_. Thus, we can assume that the number of oxygen vacancies at the TiN/ZnO interface is higher than at Pt/ZnO, that is, *D_BE_*(TiN) > *D_BE_*(Pt). This leads to the fact that the TiN/ZnO/W structure shows higher currents than the Pt/ZnO/W structure (Figure 2a,b) and hence lower resistance values *R_HRS_* and *R_LRS_* (Table 1).

The resistance *R_HRS_* for the ZnO:In/ZnO/W structure is lower than that for TiN/ZnO/W, which can be explained by the fact that the destroyed section of the conduction channel *h_gap_* for ZnO:In is smaller than that for TiN. In turn, the *R_LRS_* for the ZnO:In/ZnO/W structure is larger than that for TiN/ZnO/W, which can be explained by the fact that the diameter of the apex of the nanoscale conduction channel (*D_BE_*), due to the smaller number of In atoms compared to TiN, for ZnO:In is less than that for TiN. The large dispersion of the *R_HRS_* and *R_LRS_* of the ZnO:In/ZnO/W structure can be explained by the fact that resistive switching can occur with approximately the same probability at the ZnO:In/ZnO interface and ZnO/W (Figure 3c,d).

Resistance *R_HRS_* and *R_LRS_* for the ZnO:Pd/ZnO/W structure is significantly lower than for the other samples, despite the ∆*G* curves for Pd + O_2_ (Equations (S4) and (S6)) lying above the ∆*G* curve for Zn + O_2_ (Equation (S9)). It can be assumed that the current through the ZnO:Pd/ZnO interface is not associated with oxygen vacancies but with a rather low height of the potential barrier, because the difference between the work functions (∆*A*) for Pd and ZnO is 4.98 eV and 5.20 eV (∆*A* = 0.22 eV). For comparison, the ∆*A* for In and ZnO was approximately 1.6 eV. A small dispersion of *U_set_* and *U_res_* was observed for samples with ZnO:In and ZnO:Pd, possibly because the bottom electrode and the ZnO film in these cases have the same values of the lattice constant, which affects the stability of the resistive switching parameters. Different values of the *R_HRS_*/*R_LRS_* ratio for all samples can be explained by different values of *h_gap_* [14,15,23]. From this it follows that *h_gap_*(TiN) > *h_gap_*(Pt) > *h_gap_*(ZnO:In) > *h_gap_*(ZnO:Pd).

**Figure 4:**
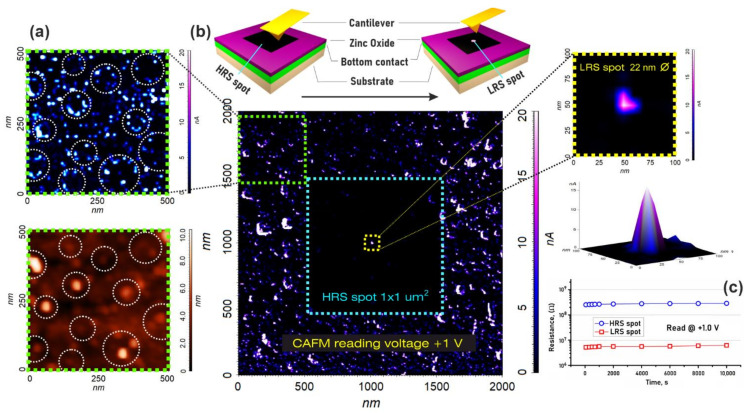
Conductive atomic-force microscopy of Al_2_O_3_/TiN/ZnO structure: (**a**)—study of the impact of morphology on the ZnO film conductivity; (**b**)—scheme and experimental results of the ZnO film switching between HRS and LRS; (**c**)—retention test.

Analysis of the experimental results obtained from the nanoscale resistive switching study showed that the current flows mainly along the grain boundaries and represents localized light dots in the CAFM (Figure 4a). Thus, we assumed that the nanoscale conduction channels of oxygen vacancies are located along the boundary of the grains. To confirm our assumption, we formed the HRS spot 1 × 1 um^2^ on the ZnO surface, and then inside it, at the grain boundary, we formed the LRS spot about 22 nm diameter (Figure 4b). An increase in time from 60 to 10^4^ s was shown to lead to an increase in resistance from 252.38 ± 18.12 MΩ to 286.21 ± 23.87 MΩ for HRS and from 5.16 ± 0.43 MΩ to 6.17 ± 0.62 MΩ for LRS (Figure 4c). The result obtained can be explained by the additional oxidation of the zinc oxide with atmospheric oxygen.

Thus, based on the obtained experimental results, it can be assumed that in our case the most suitable bottom-electrode material for forming-free nanocrystalline ZnO films is TiN. This conclusion is due to the TiN/ZnO sample having the highest *R_HRS_*/*R_LRS_* ratio, a parameter that determines the degree of multibit and hence the depth of learning of the future neuromorphic system. The structure of TiN/ZnO/W also showed acceptable values of *U_set_* = 1.9 ± 0.2 V and *U_res_* = −1.3 ± 0.5 V, which is important for creating energy-efficient elements of memristive neuromorphic systems. It should also be noted that titanium nitride and platinum are among the materials most used for RRAM prototyping, as they have a large difference in work function with ZnO (ZnO/TiN ≈ 1.53 eV, ZnO/Pt 0.85 eV), which is important to ensure a high *R_HRS_*/*R_LRS_* ratio [68,69,70].

Analysis of the obtained experimental results showed that an increase in the number of laser pulses from 1000 to 5000 leads to an increase in the thickness of the nanocrystalline ZnO film from 7.2 ± 2.5 nm to 53.6 ± 18.3 nm and the surface roughness from 2.3 ± 0.2 nm to 16.2 ± 2.1 nm (Table 2). The slowdown in the film growth rate can be explained by different growth kinetics with an increase in the number of laser pulses: in the range from 1000 to 3000 laser pulses—film growth occurs due to the diffusion of atoms along the grain boundaries (ripening and surface mobility)—and in the range from 3000 to 5000 pulses, with transition to the usual kinetics of grain growth. The increase in roughness can be explained by the intensification of the coalescence process with an increase in the thickness of the ZnO film. [71].

**Figure 5:**
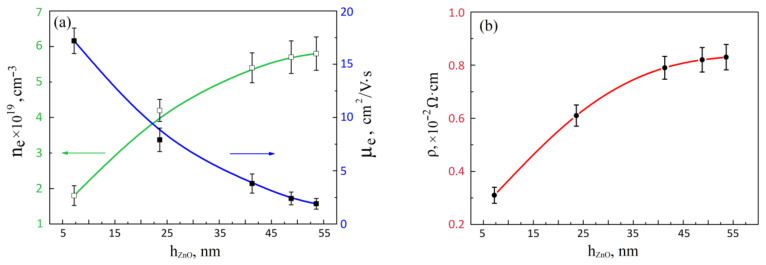
Experimental investigation of the thickness of the nanocrystalline ZnO film on electron concentration, electron mobility (**a**), and resistivity (**b**).

An analysis of the results obtained to study the effect of the thickness of the nanocrystalline ZnO film on its electrophysical parameters showed that an increase in *h_ZnO_* from 7.2 ± 2.5 nm to 53.6 ± 18.3 nm led to an increase in electron concentration from (1.8 ± 0.3) × 10^19^ cm^−3^ to (5.7 ± 0.5) × 10^19^ cm^−3^ (Figure 5a), the electron mobility from 17.2 ± 1.2 cm^2^/(V∙s) to 1.9 ± 0.5 cm^2^/(V∙s) (Figure 5a), as well as an increase in resistivity from (0.31 ± 0.03) × 10^−2^ Ω∙cm to (0.83 ± 0.04) × 10^−2^ Ω∙cm (Figure 5b). This result can be explained by the fact that both bulk conductivity and grain-boundary conductivity contribute to the total conductivity of these films. In this case, with an increase in the film thickness (as well as an increase in the grain diameter), the contribution of the current flowing through the grain volume decreases because of an increase in the distance that charge carriers must overcome from one edge of the grain boundary to its other edge [72].

**Figure 6:**
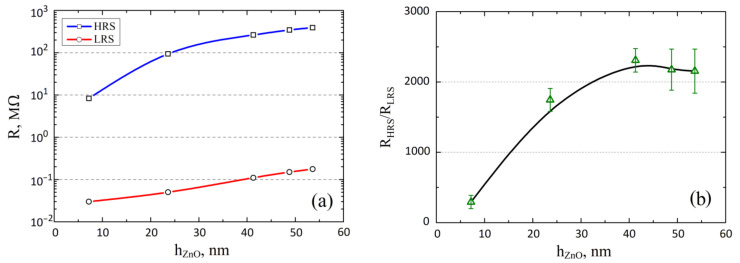
Dependence of the resistive switching parameters of the Al_2_O_3_/TiN/ZnO/W structure on the thickness of the ZnO film: (**a**)—*R_HRS_* and *R_LRS_*; (**b**)—*R_HRS_/R_LRS_*.

Analysis of experimental dependences showed that an increase in the thickness of the nanocrystalline ZnO film from 7.2 ± 2.5 nm to 53.6 ± 18.3 nm led to an increase in *R_HRS_* from 8.12 ± 0.79 MΩ to 386.71 ± 18.22 MΩ and *R_LRS_* from 0.030 ± 0.007 MΩ to 0.182 ± 0.018 MΩ, respectively (Figure 6a). In this case, the *R_HRS_*/*R_LRS_* ratio increased from 292.7 ± 94.6 to 2307.8 ± 166.4 with an increase in film thickness from 7.2 ± 2.5 nm to 41.2 ± 9.7 nm and decreased from 2307.8 ± 166.4 to 2154.7 ± 313.3 with an increase in film thickness from 41.2 ± 9.7 nm to 53.6 ± 18.3 nm (Figure 6b). One of the probable reasons for the increase in resistance of *R_HRS_* is an increase in the length of the nanoscale conduction channel gap (*h_gap_*) with an increase in the thickness of the forming-free nanocrystalline ZnO film, which is correlated with [73,74,75].

An increase in resistance *R_LRS_* can be explained by a decrease in the diameter of the apex of the nanoscale conduction channel *D_BE_* (Figure 3c) with an increase in the thickness of the nanocrystalline ZnO film. If we follow this hypothesis, then the decrease in *R_HRS_*/*R_LRS_* can be explained by the fact that the *D_BE_*/*h_gap_* ratio for films with a thickness from 7.2 ± 2.5 nm to 41.2 ± 9.7 nm is greater than for films with a thickness of 48.8 ± 15.0 nm and 53.6 ± 18.3 nm. The large dispersion of *R_HRS_*/*R_LRS_* for films with a thickness of 48.8 ± 15.0 nm and 53.6 ± 18.3 nm can be explained by an increase in the nonuniformity of oxygen vacancy generation and recombination in most of the oxide film due to a decrease in the electron concentration with an increase in film thickness (Figure 5a), due to which the *h_gap_* varies widely with each new switching cycle.

**Figure 7:**
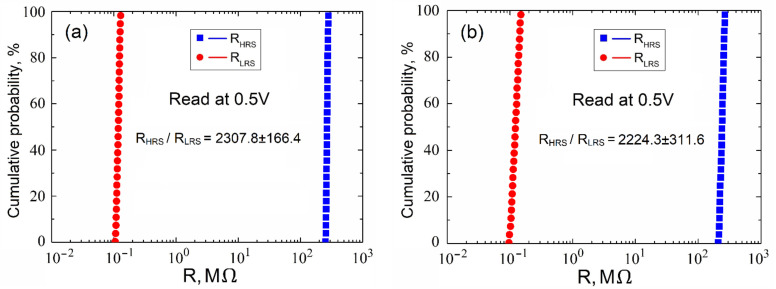
Investigation of resistive switching in the forming-free nanocrystalline ZnO film (h_ZnO_ = 41.2 ± 9.7 nm): (**a**)—at the same point on the film surface (endurance) and (**b**)—at different points on the film surface (homogeneity).

Analysis of the obtained experimental results obtained for the resistance values (Figure 7) (here, to avoid confusion, the resistances at one point on the surface of the ZnO film are designated *R_HRSp_* and *R_LRSp_* and at different points as *R_HRSs_* and *R_LRSs_*) showed that an increase in the thickness of the film from 7.2 ± 2.5 nm to 53.6 ± 18.3 nm leads to an increase in the resistances *R_HRSp_* and *R_LRSp_* from 8.12 ± 0.79 MΩ to 386.71 ± 18.22 MΩ and from 0.030 ± 0.007 MΩ to 0.182 ± 0.018 MΩ, respectively (Table 3). The resistances of *R_HRSs_* and *R_LRSs_* increased from 7.93 ± 0.93 MΩ to 378.81 ± 38.82 MΩ and from 0.035 ± 0.012 MΩ to 0.182 ± 0.053 MΩ, respectively. The *R_HRSp_*/*R_LRSp_* and *R_HRSs_*/*R_LRSs_* ratios increased from 292.7 ± 94.6 to 2154.7 ± 313.3 and from 267.1 ± 118.1 and 2342.1 ± 895.3, respectively. The large dispersion of R*_HRS_* and R*_LRS_* values can be explained by the inhomogeneity of the morphology of nanocrystalline ZnO films (Figure 1), leading to an uneven concentration of oxygen vacancies in different areas of the nanocrystalline ZnO film.

Therefore, for the fabrication of memristive elements of neuromorphic systems from the studied samples, the most preferable structure was TiN/ZnO/W with the forming-free nanocrystalline ZnO thickness 41.2 ± 9.7 nm, which has a sufficiently high ratio *R_HRS_/R_LRS_* = 2307.8 ± 166.4 and has the best, compared to other samples, endurance and homogeneity.

**Figure 8:**
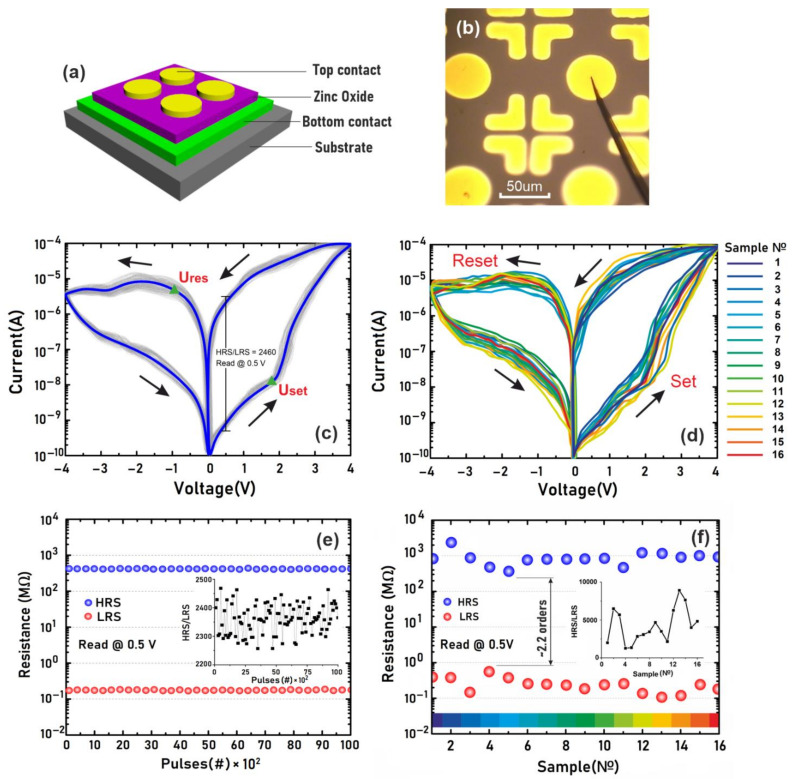
Investigation of resistive switching in the Al_2_O_3_/TiN/ZnO/TiN memristive structures: (**a**)—conceptualization model; (**b**)—photomicrograph; (**c**)—current-voltage characteristics of a single device; (**d**)—current-voltage characteristics of 16 different devices; (**e**)—endurance test of a single device; (**f**)—reproducibility test.

Analysis of the results of the study of the influence of the top electrode on the effect of resistive switching in the structure Al_2_O_3_/TiN/ZnO/TiN (Figure 8a,b) showed that the structure shows stable resistive switching with *U_set_* = 1.6 ± 0.2 V and *U_res_* = −0.8 ± 0.3 V (Figure 8c). The endurance test showed that *R_HRS_* is 417.52 ± 3.57 kΩ and *R_LRS_* is 0.17 ± 0.03 kΩ (Figure 8e). It was shown that the *R_HRS_*/*R_LRS_* ratio was about 2460 at a 0.5 V read voltage. A study of the device-to-device resistive switching showed that the *R_HRS_*/*R_LRS_* ratio was higher than two orders of magnitude (Figure 8d,f). Thus, the use of the top electrode made it possible to reduce the switching voltages *U_set_* and *U_res_* and to increase the *R_HRS_*/*R_LRS_* ratio. The results can be explained by the exclusion of atmospheric oxygen influence, which penetrates the ZnO film during resistive switching and creates additional energy barriers in the Al_2_O_3_/TiN/ZnO structure.

**Figure 9:**
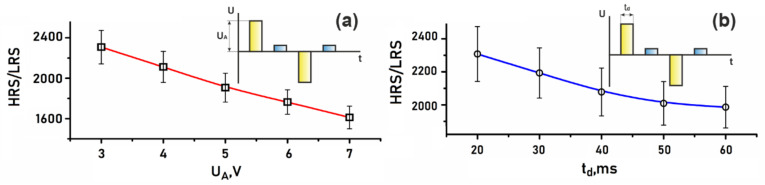
Experimental investigations of dependences of the RHRS/RLRS ratio on pulse: (**a**)—amplitude; (**b**)—duration.

It was shown that in the Al_2_O_3_/TiN/ZnO/TiN memristive structure, an increase in *U_A_* from 2 V to 7 V led to a decrease in the *R_HRS_/R_LRS_* ratio from 2307.8 ± 166.4 to 1612.3 ± 112.4 (Figure 9a), and an increase in the *t_A_* from 20 ms to 60 ms led to a decrease in the *R_HRS_/R_LRS_* ratio from 2307.8 ± 166.4 to 1986.7 ± 126.8 (Figure 9b). The results can be explained by the increase in the concentration of oxygen vacancies and the nanoscale conducting channel diameters D_TE_ and D_BE_ (Figure 3b,c) in the nanocrystalline ZnO film with an increase in *U_A_* and *t_A_*.

## 4. Conclusions

The article presented the novel results of experimental studies of the bottom material and the impact of size on resistive switching in the nanocrystalline ZnO film fabricated by pulsed laser deposition. It was demonstrated that the nanocrystalline ZnO film exhibits a non-linear bipolar effect of forming-free resistive switching. The Ellingham diagrams for TiN + O_2_, Pt + O_2_, In +O_2_, and Pd + O_2_ were built. The sample with Pt showed the highest resistance values *R_HRS_* and *R_LRS_* and the highest value of *U_set_* = 2.7 ± 0.4 V. Samples with ZnO:In and ZnO:Pd bottom electrodes showed the lowest *U_set_* and *U_res_* values. It was shown that in our case, the most suitable bottom-electrode material for forming-free nanocrystalline ZnO films was TiN, since it had the highest *R_HRS_*/*R_LRS_* ratio, a parameter that determines the degree of multibit, and hence the depth of learning of the future neuromorphic system. Furthermore, the structure of TiN/ZnO/W demonstrates acceptable values of *U_set_* = 1.9 ± 0.2 V and *U_res_* = −1.3 ± 0.5 V, which is important to create energy-efficient elements of memristive neuromorphic systems.

It was shown that an increase in the number of laser pulses from 1000 to 5000 leads to an increase in the thickness of the nanocrystalline ZnO film and surface roughness. An analysis of electrophysical parameters showed that an increase in the thickness of the ZnO film from 7.2 ± 2.5 nm to 53.6 ± 18.3 nm leads to an increase in electron concentration from (1.8 ± 0.3) × 10^19^ cm^−3^ to (5.7 ± 0.5) × 10^19^ cm^−3^, a decrease in electron mobility from 17.2 ± 1.2 cm^2^/(V∙s) to 1.9 ± 0.5 cm^2^/(V∙s), and an increase in resistivity from (0.31 ± 0.03) × 10^−2^ Ω∙cm to (0.83 ± 0.04) × 10^−2^ Ω∙cm.

It was shown by the method CAFM that the in nanocrystalline ZnO films’ current flows mainly along the grain boundaries and that the nanoscale conduction channels are located along the boundary of the grains.

The obtained experimental results showed that an increase in the thickness of the nanocrystalline ZnO film from 7.2 ± 2.5 nm to 41.2 ± 9.7 nm leads to an increase in the *R_HRS_/R_LRS_* ratio and decreases the *R_HRS_/R_LRS_* ratio with an increase in film thickness from 41.2 ± 9.7 nm to 53.6 ± 18.3 nm. Analysis of the results obtained for the endurance and the homogeneity tests showed that the ratios of *R_HRSp_/R_LRSp_* and *R_HRSs_/R_LRSs_* increased from 292.7 ± 94.6 to 2154.7 ± 313.3 and from 267.1 ± 118.1 and 2342.1 ± 895.3, respectively. The large dispersion of the values of *R_HRSs_* and *R_LRSs_* can be explained by the inhomogeneity of the morphology of the nanocrystalline ZnO films, which leads to an uneven concentration of oxygen vacancies over the surface.

Therefore, it was shown that among the samples considered in this work, the TiN/ZnO/W structure with the forming-free nanocrystalline ZnO thickness 41.2 ± 9.7 nm is preferable for the manufacture of memristive neuromorphic systems due to the highest values of the *R_HRS_/R_LRS_* ratio and the relatively low values of *U_set_* and *U_res_*. It was shown that the use of the top electrode made it possible to reduce the switching voltages *U_set_* and *U_res_* and to increase the *R_HRS_*/*R_LRS_* ratio.

The results obtained can be used in the manufacturing of resistive-switching nanoscale devices for neuromorphic computing based on the forming-free nanocrystalline ZnO oxide films.

## Figures and Tables

**Table 1:** Resistive-switching parameters of the forming-free nanocrystalline ZnO films for different bottom-electrode materials.

Bottom-Electrode Material	TiN	Pt	ZnO:In	ZnO:Pd
*R_HRS_*, MΩ	276.11 ± 8.43	1141.37 ± 13.24	61.38 ± 2.54	0.67 ± 0.01
*R_LRS_*, MΩ	0.120 ± 0.005	1.310 ± 0.003	0.231 ± 0.032	0.041 ± 0.002
*R_HRS_/R_LRS_*	2307.8 ± 166.4	871.3 ± 12.1	272.5 ± 48.7	16.4 ± 1.1
*U_set_*, V	1.9 ± 0.2	2.7 ± 0.4	2.3 ± 0.2	1.2 ± 0.1
*U_res_*, V	−1.3 ± 0.5	−2.2 ± 0.6	−2.7 ± 0.3	−1.3 ± 0.1

**Table 2:** PLD regime, experimental film thickness, and surface roughness of the forming-free nanocrystalline ZnO.

No. Sample	1	2	3	4	5
Substrate temperature, °C			500		
Number of laser pulses	1000	2000	3000	4000	5000
Laser pulse repetition rate, Hz			10		
Oxygen pressure, mTorr			0.5		
Film thickness (*h_ZnO_*), nm	7.2 ± 2.5	23.6 ± 6.7	41.2 ± 9.7	48.8 ± 15.0	53.6 ± 18.3
Surface roughness (*R_a_*), nm	2.3 ± 0.2	5.5 ± 1.2	8.1 ± 1.6	13.1 ± 1.9	16.2 ± 2.1

**Table 3:** Experimental investigations of the resistance of Al_2_O_3_/TiN/ZnO/W at the same point (*R_HRSs_* and *R_LRSs_*) and at different points on the forming-free nanocrystalline ZnO film surface (*R_HRSp_* and *R_LRSp_*).

No. Sample	1	2	3	4	5
*R_HRSp_*, MΩ	8.12 ± 0.79	104.22 ± 4.52	276.11 ± 8.43	305.12 ± 13.11	386.71 ± 18.22
*R_LRSp_*_,_ MΩ	0.030 ± 0.007	0.060 ± 0.003	0.120 ± 0.005	0.142 ± 0.013	0.182 ± 0.018
*R_HRSs_*, MΩ	7.93 ± 0.93	89.31 ± 12.7	250.54 ± 15.52	328.26 ± 39.41	378.81 ± 38.82
*R_LRSs_*, MΩ	0.035 ± 0.012	0.061 ± 0.002	0.114 ± 0.009	0.148 ± 0.026	0.182 ± 0.053
*R_HRSp_/R_LRSp_*	292.7 ± 94.6	1745.1 ± 162.6	2307.8 ± 166.4	2175.4 ± 291.4	2154.7 ± 313.3
*R_HRSs_/R_LRSs_*	267.1 ± 118.1	1472.5 ± 256.4	2224.3 ± 311.6	2336.8 ± 676.8	2342.1 ± 895.3

## Supplementary Materials

The following are available online at https://www.mdpi.com/article/10.3390/nano12030455/s1, Figure S1: Experimental studies of the forming-free nanocrystalline ZnO film thickness: (a)—AFM-image; (b)—AFM cross section along the white line in (a); (c)—3D AFM-image; (d)—height histogram; Equations (S1)–(S9): Reduction reaction at the interface between the electrode and the forming-free nanocrystalline ZnO film.
