# Supplementary material for: Nanoscale-Resistive Switching in Forming-Free Zinc Oxide Memristive Structures
Source: Nanomaterials (Basel). 2022 Jan 28;12(3):455. doi: 10.3390/nano12030455 (PMC8838399; doi:10.3390/nano12030455)
Supplement: Supplementary file 1 [file nanomaterials-12-00455-s001.zip › nanomaterials-1530140-supplementary.pdf]

nanomaterials

Supplementary Materials

Nanoscale-Resistive Switching in Forming-Free Zinc Oxide Memristive Structures

Roman V. Tominov [1], Zakhar E. Vakulov [3], Nikita V. Polupanov [4], Aleksandr V. Sanko [1], Vadim I. Avilov [2], Oleg A. Ageev [2] and Vladimir A. Smirnov [1,*]

[1] Department of Radioelectronics and Nanoelectronics, Institute of Nanotechnologies, Electronics and Electronic Equipment Engineering, Southern Federal University, Taganrog 347922, Russia; tominov@sfedu.ru (R.V.T.); avsaenko@sfedu.ru (A.V.S.); vasmirnov@sfedu.ru (V.A.S)
[2] Department of Micro- and Nanoelectronics, Institute of Nanotechnologies, Electronics and Electronic Equipment Engineering, Southern Federal University, Taganrog 347922, Russia; avilovvi@sfedu.ru (V.I.A.); ageev@sfedu.ru (O.A.A.)
[3] Federal Research Centre the Southern Scientific Centre of the Russian Academy of Sciences, Rostov-on-Don 344006, Russia; vakulov@ssc-ras.ru
[4] Laboratory of Functional Nanomaterials Technology, Southern Federal University, Taganrog 347922, Russia; npolupanov@sfedu.ru
* Correspondence: vasmirnov@sfedu.ru

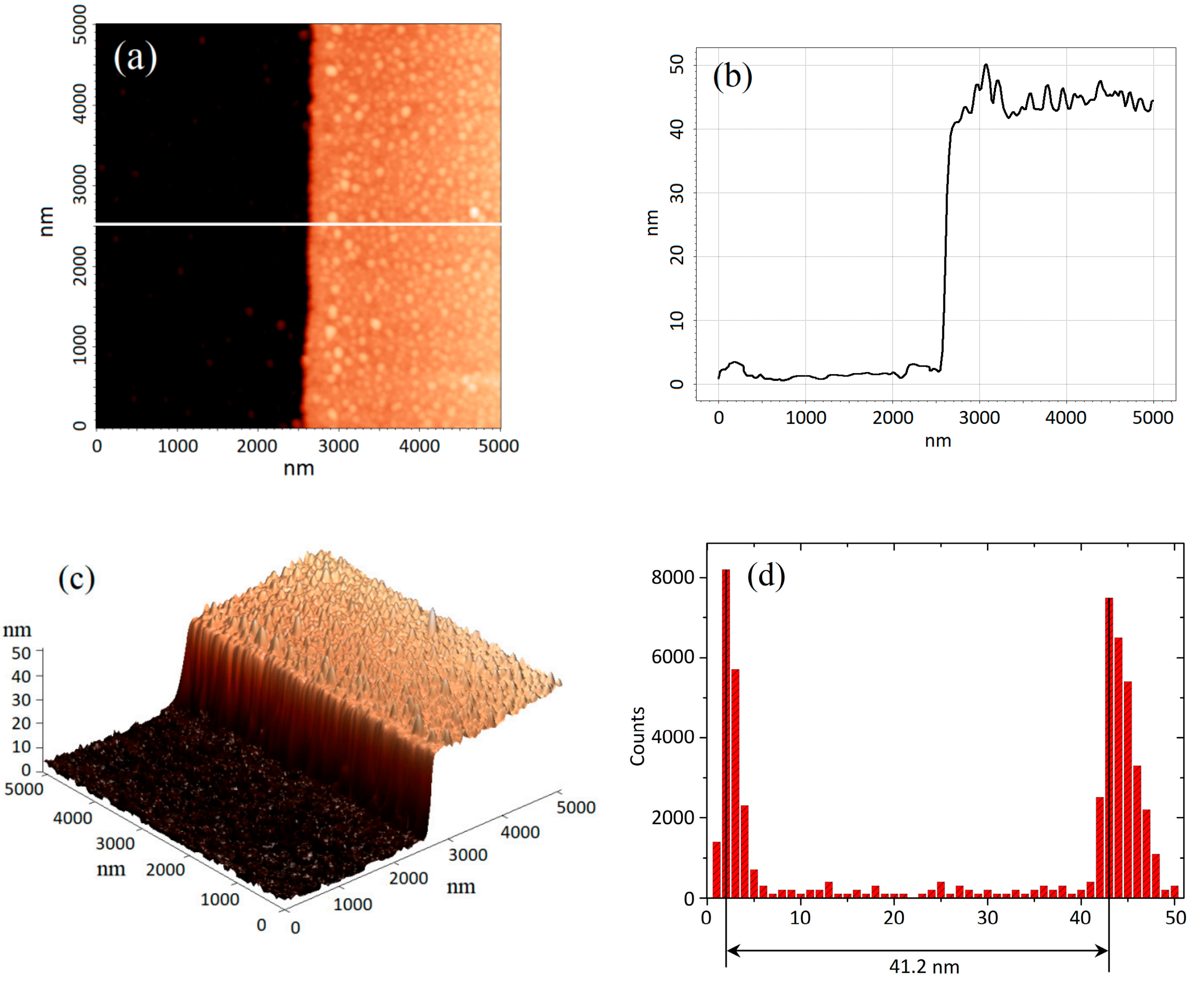

Figure S1. Experimental studies of the forming-free nanocrystalline ZnO film thickness: (**a**) – AFM-image; (**b**) – AFM cross section along the white line in (**a**); (**c**) – 3D AFM-image; (**d**) – height histogram.

$$2TiN + 2O_2 \rightarrow 2TiO_2 + N_2 \quad \text{(S1)}$$
$$2Pt + O_2 \rightarrow 2PtO \quad \text{(S2)}$$
$$Pt + O_2 \rightarrow PtO_2 \quad \text{(S3)}$$
$$4In + 3O_2 \rightarrow 2In_2O_3 \quad \text{(S4)}$$
$$2Pd + O_2 \rightarrow 2PdO \quad \text{(S5)}$$
$$Pd + O_2 \rightarrow PdO_2 \quad \text{(S6)}$$
$$2W + 3O_2 \rightarrow 2WO_3 \quad \text{(S7)}$$
$$2W + O_2 \rightarrow 2WO \quad \text{(S8)}$$
$$2Zn + O_2 \rightarrow 2ZnO \quad \text{(S9)}$$

Equations S1–S9. Reduction reaction at the interface between the electrode and the forming-free nanocrystalline ZnO film.
